# Progressive Cachexia: Tuberculosis, Cancer, or Thyrotoxicosis? Disease-Directed Therapy and Atypical Courses of Autoimmune and Malignant Thyroid Diseases in a High Specialization Era: Case-Control Study with a Critical Literature Review

**DOI:** 10.3390/biomedicines12122722

**Published:** 2024-11-28

**Authors:** Przemyslaw Zdziarski, Zbigniew Sroka

**Affiliations:** 1 Lower Silesian Center, 53-413 Wroclaw, Poland; 2 PRION Private Research Institute of Nature, 50-385 Wroclaw, Poland; 3 Department of Pharmacognosy and Herbal Medicines, Wroclaw Medical University, 50-367 Wrocław, Poland; zbigniew.sroka@umw.edu.pl

**Keywords:** thyroid cancer, BRAFV600E mutation, autoimmune thyroiditis, paraneoplastic syndrome, mycobacterial disease, cachexia, myopathy, nuclear factor kappa-light-chain-enhancer of activated B cells (NF-κB), calcium homeostasis, statistical bias, precision medicine

## Abstract

**Background.** Critical and progressive cachexia may be observed in numerous medical disciplines, but in patients with various diseases, several pathways overlap (endocrine, inflammatory and kidney diseases, heart failure, cancer). **Methods.** Unlike numerous cohort studies that examine thyroid cancer and risk factors, a different method was used to avoid bias and analyze the sequence of events, i.e., the pathway. A case-control analysis is presented on patients with initial immune-mediated thyroiditis complicated by cachexia, presenting pulmonary pathology coexisting with opportunistic infection, and ultimately diagnosed with cancer (TC—thyroid cancer, misdiagnosed as lung cancer). **Results.** Contrary to other patients with lung cancer, the presented patients were not active smokers and exclusively women who developed cachexia with existing autoimmune processes in the first phase. Furthermore, the coexistence of short overall survival without cancer progression in the most seriously ill patients, as well as correlation with sex (contrary to history of smoking) and predisposition to mycobacterial disease, are very suggestive. Although we describe three different autoimmune conditions (de Quervain’s, Graves’, and atrophic thyroiditis), disturbances in calcium and metabolic homeostasis, under the influence of hormonal and inflammatory changes, are crucial factors of cachexia and prognosis. **Conclusions.** The unique sequence sheds light on immune-mediated thyroid disease as a subclinical paraneoplastic process modified by various therapeutic regimens. However, it is also associated with cachexia, systemic consequences, and atypical sequelae, which require a holistic approach. The differential diagnosis of severe cachexia, adenocarcinoma with pulmonary localization, and tuberculosis reactivation requires an analysis of immunological and genetic backgrounds. Contrary to highly specialized teams (e.g., lung cancer units), immunotherapy and general medicine in aging populations require a multidisciplinary, holistic, and inquiring approach. The lack of differentiation, confusing biases, and discrepancies in the literature are the main obstacles to statistical research, limiting findings to correlations of common factors only. Time-lapse case studies such as this one may be among the first to build evidence of a pathway and an association between inflammatory and endocrine imbalances in cancer cachexia.

## 1. Introduction

Hormone-derived signals are among the most intensively studied factors in carcinogenesis. However, the pathological hypersecretion of several hormones is an area of endocrinology, despite its cross-talk with cancer and immunity.

Furthermore, immunoendocrinopathy shows the balance between the pro-cancer (hormonal) and anticancer (immune) pathways. In this context, organ-specific autoimmunity, such as autoimmune thyroid disease (AITD), may be a good model for looking for more specific immunotherapies than systemic immune checkpoint blockade. The role of the local niche seems to be underestimated, and research is mainly conducted on the intestinal microbiota [1]. However, the pulmonary microbiome is one additional pathogenetic factor (extremely variable) that affects overall survival in various ways [2]. Furthermore, immunity in patients with lung cancer may be difficult because most microbial antigens from mycobacteria, fungi, and viruses involve lymphocytes, sometimes inducing lymphoproliferative disease. These microbial antigens can also induce a cytokine storm and “spontaneous” regression [3]. Currently, the research includes retrospective studies on patient with advanced thyroid cancer (TC) only (the precancerous stage is omitted). Previous history, including AITD, is usually briefly described, taking into account only TSH levels, antibodies against the TSH receptor (TRAB), and Graves’ disease (GD). The higher incidence of thyroid carcinoma in patients with GD has been studied in many cohort studies, but the crucial issue is the cause-and-effect relationship, not a statistical one. The initial paradigm viewed hyperthyroidism as a protective mechanism due to suppressed hormonal signals (i.e., TSH). However, further statistical analyses have shown a higher coexistence of Graves’ disease (GD) with thyroid cancer. Despite this, high GD prevalence in thyroid cancer is only a statistical link (pathways are not described) [4]. For example, when TC coexists with GD, the immunogenetic background and the role of autoimmunity in TC are not explained [5]. TSH, TRAB, and manifestations are described only qualitatively. Therefore, in such publications, the co-occurrence rather than the mutual influence of such processes is described. Thus, the correlation between GD and TC is controversial.

For clinical immunologists and patient-centered care, the crucial question is the following: what is the cause, and what is the effect? Patient time-lapse analysis may be a good tool for this observation. A very intensive immune response may be the cause of proliferative disease (a precancerous state) but—on the other hand—also provide very beneficial immunity against tumor-specific antigens, such as thyroglobulin (TG).

Interestingly, most TCs have a small tumor size (with about 50% being below 1 cm) [6] without stroma. However, an immune response against thyroid antigens may be beneficial, since the most common adverse effect of immunotherapy (e.g., with interferons or immune checkpoint blockade) is autoimmune thyroiditis [7,8]. Now we know that the immune response has a critical surveillance role against cancer, but immunity against cancer cells usually cross-reacts with host cells, with severe outcomes. Therefore, autoimmunity against organ-/tissue-specific antigens is a good model for evaluating immunotherapy-induced adverse drug reactions to assess the risk–benefit ratio.

We looked for cases of autoimmune endocrinopathy in the first stage, with presentation of cancer in the next step, coexisting with long-term infectious inflammation. (e.g., tuberculosis).

The new methodology, as precisely described in Section 2, helped us to find temporal connections and pathways.

## 2. Materials and Methods

### 2.1. Material

**Figure 1:**
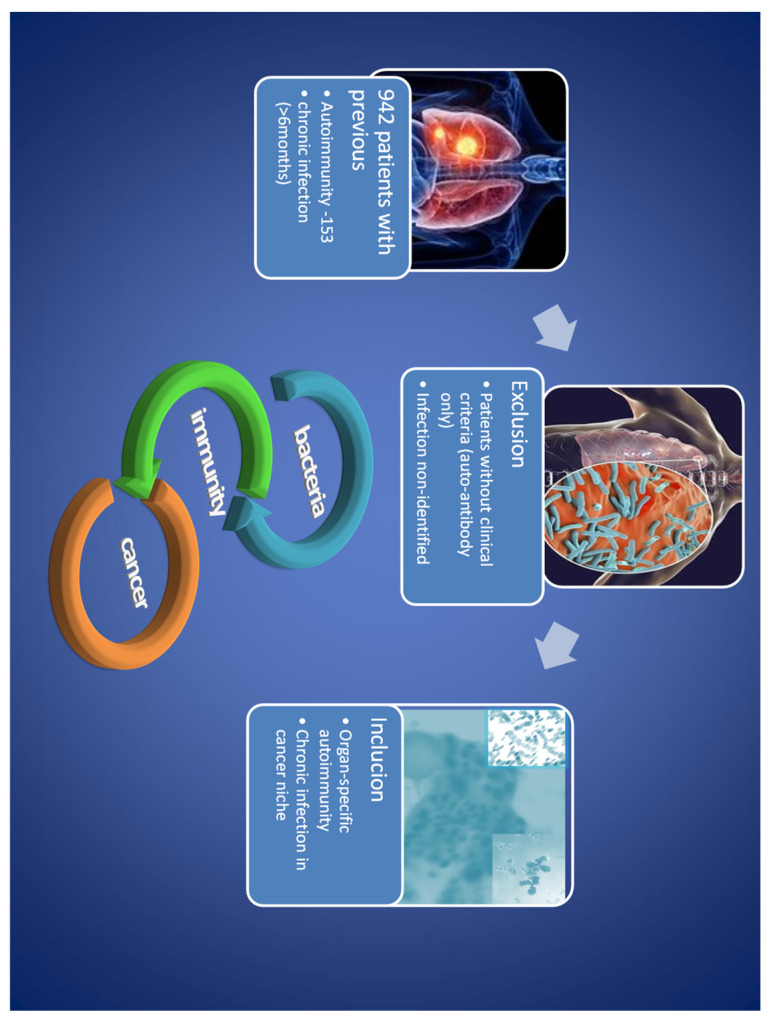
Initial patient selection. After initial selection, a small amount of patients was qualified, but contrary to most retrospective analyses of patients with thyroid cancer, in our clinical model, AITD preceded oncogenesis and may be with different types of AITD (i.e., de Quervain thyroiditis, Graves’ disease, Hashimoto/atrophic thyroiditis).

The observational study comprised the history of 942 cancer patients described previously [9]. Thus, the data from patients with mis-differentiation, without clinical and laboratory criteria (i.e., symptoms with microbiological and serological confirmation), were not included in the study. Consequently, most of the patients were disqualified (Figure 1).

Only patients diagnosed with well-documented autoimmunity (clinical criteria) and chronic (≥6 months) infection were approached for an interview. The patients were a heterogeneous group comprising various stages and development of inflammatory and neoplastic processes. To exclude common inflammatory complications of cancer or chemotherapy, we included only patients with previously diagnosed inflammatory disease.

Secondly, the patients with severe and systemic autoimmunity usually were treated with immunosuppressive drugs (e.g., corticosteroids). Therefore, the study yielded confusing results. Patients with local organ-specific autoimmunity were not subject to mandatory immunosuppressive therapy (e.g., AITD). This allows, on the one hand, to look for less disturbing immunotherapy (local antigens) and, on the other hand, to monitor the scale of the immune response with specific biochemical parameters.

### 2.2. Methods

#### 2.2.1. Time-Lapse Data Collection

**Figure 2:**
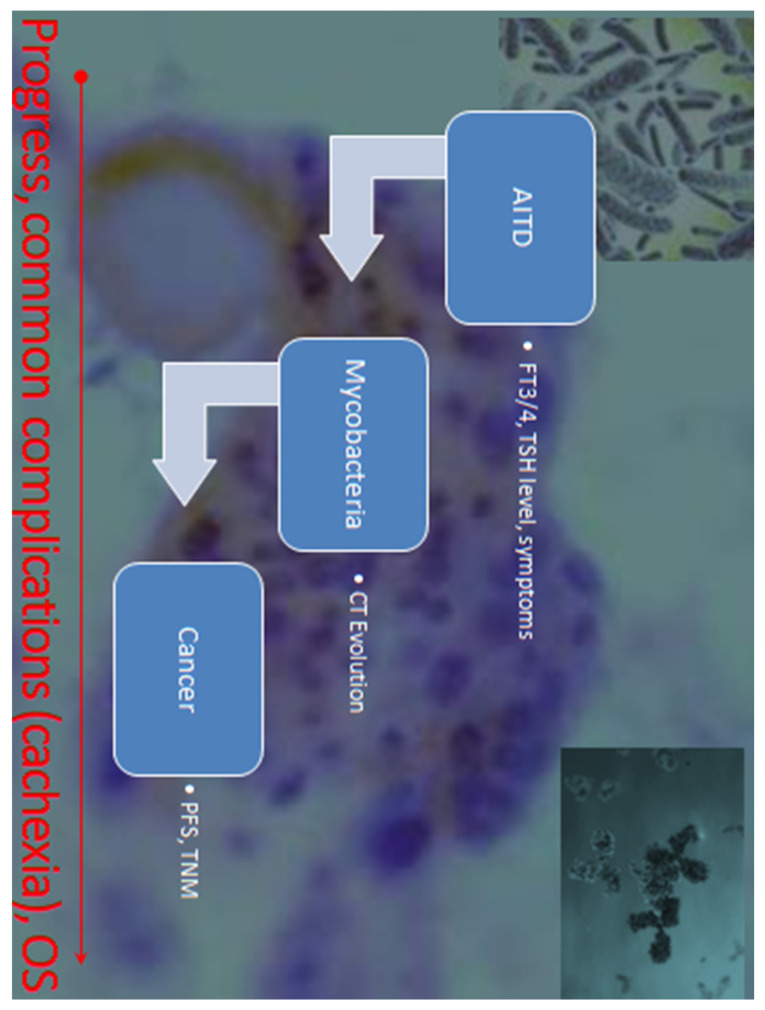
Flowchart of clinical data collection and time-lapse analysis. Patients with autoimmune thyroid disease (AITD) were the starting point. The case-control study includes patient histories with well-characterized and differentiated autoimmune thyroid disease (AITD) complicated with infectious and neoplastic processes. TC was the sixth cancer in women; it was not observed in men. However, this could be apparent because the initial group consisted of patients with autoimmunity, which is more common in women with no difference between multiparous and childless. Comparing our AITDs where hyperthyroidism, hypothyroidism, or both occurred at different times, no clear effect of hypothyroidism and elevated TSH can be seen. AITD—autoimmune thyroid disease, PFS—progression-free survival, OS—overall survival, TSH—thyroid-stimulating hormone, FT3—free triiodothyronine, FT4—free thyroxine, CT—computer tomography.

We analyzed selected clinical parameters most representative of the three diseases (imaging, biochemical, serological tests) linking them to the parallel progression (progression/regression) of pathological processes and the impact on overall survival. The flowchart of the systematic clinical observation and data collection is presented in Figure 2.

#### 2.2.2. Laboratory Analysis

Real-time polymerase-chain reaction (RT-PCR) for the MTB complex was performed using the MTB/RIF (Xpert) test, GeneXpert^®^ (Cepheid, Sunnyvale, CA, USA), which was endorsed by the World Health Organization. The lower sensitivity limit is 30 and 12 CFU/mL for M. tuberculosis and M. bovis, respectively.

In IHC analysis, the antibody clones Dako, 8G7G3/1and SP 263 were used for TTF-1 and PD-L1 expression respectively. Although positive labeling for TTF-1 can occur in tumors from other sites, the monoclonal antibodies used are characterized by the highest specificity among those commercially available, and TTF-1 expression on other cancers (except NSCLC and TC) does not exceed several percentage points [10,11]. For differentiation, where sufficient tumor samples were obtained, molecular testing was performed. Other organs showing potential TTF-1 expression, such as the intestines, bile ducts, and pancreas, were also examined with diagnostic imaging.

Next-generation sequencing (NGS) was performed using the FusionPlex^®^ Lung Kit (Integrated DNA Technologies, Inc. (Coralville, IA, USA) according to the producer’s description (Archer ref. SK0133) on MiSeqDx equipment (Illumina San Diego, CA, USA) and Archer Analysis pipeline version: 7.2.1-1 software. Scope of study: SNV variants and indels: KRAS (exons 2, 3), BRAF (exon 15), ALK (exons 22, 23, 25), RET (exons 15, 16), ROS1 (exon 38), EGFR (exons 18, 19, 20, 21); fusion variants/deletions and duplications of exons: ALK, BRAF, EGFR, MET, RET, ROS1, FGFR1, FGFR2, FGFR3, NRG1, NTRK1, NTRK2, NTRK3; loss of exon 14 of the MET gene (exon 14 skipping).

## 3. Results

This case-control study describes the temporal connection of various processes, i.e., the best documented cases of AITD course with various infectious and oncological complications and outcomes with overall survival (OS) (Figure 2).

The time-lapse analysis of mutual biochemical, endocrine, and immunological parameters is presented in Table 1. Comparing our AITDs where hyperthyroidism, hypothyroidism, or both occur at different times, no clear effect of hypothyroidism and elevated TSH on TC and outcome can be seen. The malabsorption, tumor markers and signs of other metastatic TTF-1 + cancers (colorectal, pancreatic, ovarian, and bladder carcinomas) were not observed.

The first woman (BMI 15 kg/m^2^) with initial osteopenia (received vitamin D up to 4000 IU per day) was admitted to the outpatient clinic. Due to tachycardia and hypertension, she was treated with beta-blockers, but the symptoms, weakness and fatigue were becoming worse. The patient observed severe body weight loss (6.5 kg over 6 months). The dispone was observed, and an X-ray of the chest showed small nodules. Tuberculosis was initially diagnosed because of the positive QuantiFERON result and epidemiologic data. After CT, the mediastinal lymphadenopathy and residual pleural effusion were observed. She received corticosteroids and underwent bronchoscopy. The bronchoalveolar examination showed cancerous cells, identified as non-small cell lung cancer (NSCLC), with positive thyroid transcription factor-1 (TTF-1) expression (i.e., lung adenocarcinoma). The small amount of cancer cells was not enough for further analysis. The patient was qualified for sequential chemoradiotherapy. After the first course, the patient deteriorated, and significant hyperthyroidism was observed in spite of therapy with thiamazole. Bilateral pleural effusion and atrial fibrillation developed, and symptoms progression, especially severe cachexia with a decrease in the quality of life, were observed. In the next step, the fine-needle aspiration biopsy (FNAB) of the thyroid confirmed the cancerous cells with TTF-1 expression. The bone scintigraphy was negative for bone involvement. Due to the coexistence of cardiac as well systemic symptoms (i.e., hypocalcemic tetany, cachexia), the woman was qualified for best supportive therapy (BST). Tuberculosis reactivation was observed later with unfavorable outcome.

The retrospective analysis of endocrine status revealed previous low TSH (initially 4 years before). Despite anti-thyroid peroxidase (anti-TPO 1:320) as well as anti-thyroglobulin titer (anti-TG 1:1280), the GD was not diagnosed, because the thyroid-stimulating antibodies associated with GD (TSA, i.e., activator TRAb) were not observed. The patient occasionally received thyroid hormone from the endocrinologist (transient hypothyroidism with high TSH was observed later).

Self-limiting inflammatory thyroiditis (de Quervain) was finally diagnosed because of transilient FT3/4 fluctuation, granulomatous disease, and a constellation of antibodies against anti-thyroid antigens. Radioiodine uptake was not tested.

The second 71-year-old woman was admitted to the hospital due to fatigue, cough, a severe decrease in body weight (4 kg within 2 months; BMI 13.4), hypotension, and respiratory depression with hypoxia. The initial symptoms were somnolence/sleepiness, nausea, myoclonus, and reduced renal function.

In past history, the woman was diagnosed with hypertension and had suffered from pneumonia 10 years earlier. She was also treated with radioactive iodine therapy—a safe and effective procedure for her GD hyperthyroidism (with TSA above 1:1280) —3 years before admission. For a long time, she was in good condition, and she received antihypertensive drugs and thyroid hormone supplementation. Laboratory analysis revealed a high level of FT4 = 22 pmol/L (Normal 9–19), TSH below the detection limit (i.e., 6 μIU/mL), and low calcium level Ca++ = 1.12 mmol/L.

Chest CT revealed numerous nodules in the lung apexes and a left-sided infiltration in the middle part of the lower lobe (8 × 5 cm). After bronchoscopy, bronchoalveolar lavage (BAL) showed firstly positive bacterioscopy and polymerase chain reaction (PCR) determining the Mycobacterium tuberculosis complex (the result was confirmed by mycobacterial culture later). The standard anti-tuberculosis therapy was started. In the following weeks, the patient received medications but required supportive therapy (oxygen, anti-swelling, and anti-inflammatory pain medications).

Histopathological examination revealed the presence of cancer cells in the bronchial specimen. Cancerous cells were identified as NSCLC. Further examinations showed positive TTF-1 and programmed cell death-ligand 1 (PD-L1) expression in 40% of cells.

Magnetic resonance imaging of the head showed the presence of a focus (about 2 cm) in the right hemisphere of the brain (the patient did not give consent to surgery).

NSCLC (adenocarcinoma) in the T4N2M1b stage was disqualified from palliative radiotherapy due to severe progressive cachexia, somnolence, and myoclonus. The woman died shortly afterwards (3 months later) receiving the antituberculosis drug and BST).

Unfortunately, the genetic background in NGS showed no mutation in EGFR, in KRAS, nor in ALK and ROS1 rearrangement, but it did in BRAFV600E mutation, i.e., T > A (V600E).

The third, a 69-year-old woman, not an active smoker, previously suffering from hypertension, long-term overweight (BMI = 26.64), and osteoporosis, and she was referred to a pulmonologist due to cough, shortness of breath, and fatigue. In the initial period, she took glucocorticosteroids (gks) (methylprednisolone 4–16 mg/day) with improvement. CT scan revealed emphysematous bubbles in the apical and subapical segments as well as numerous small (2–3 mm) nodules throughout the lungs. There was no lymphadenopathy detected. After gks dose reduction (to 4 mg), a temporary deterioration was observed. After a year, the patient lost 6 kg in 5 months (despite insulin resistance, hyperglycemia, and glycosuria). The TB-QuantiFERON test (QFT) showed a moderately positive (actually borderline) result.

Another CT scan revealed a clear two-centimeter tumor in the right lung (segment 2) with numerous projections and a bright center. No cancer cells or acid-fast bacilli were detected in BAL and brush biopsy. The mycobacterial culture was also negative.

The woman was qualified for thoracic surgery. In PET scanning, before the procedure, an irregular soft-tissue mass of several centimeters with deep decay was observed in segment 2 on the right side (SUV = 8.4). No lymphadenopathy was detected nor significant metabolic activity of the lymph nodes (neck nor chest).

No attention was paid to the image of the thyroid gland. However, PET also showed a small thyroid gland with an approximately 10 mm lesion in the right lobe (SUV = 5.9).

Video-assisted thoracic surgery (VATS) was performed, including right upper lobectomy and systemic lymphadenectomy (without complications).

After the lobectomy, the patient was referred to an endocrinologist. Ultrasound examination of the thyroid gland and verification were performed.

In both lobes, there were several merging hypoechoic foci of irregular shape, the largest of them in the right lobe, measuring 13.6 × 9.8 mm with significant vascularization. There were no enlarged lymph nodes along the large cervical vessels. The patient had an inconclusive FNAB result. The preparations were of extremely low cellularity: no follicular cells in the left lobe, scattered thyrocytes in the right lobe, colloidal content, and inflammatory cells. The image was consistent with atrophic thyroiditis but local atypia, lymphoid cells, and signs of cytotoxicity were observed. It corresponds with hormonal status and serology (Table 1).

Interestingly, histopathological examination showed no cancer nor typical granuloma, but massive caseous necrosis. Immunohistochemistry showed several acid-fast bacilli in necrotic mass. The tuberculosis was diagnosed taking into account the CT, QFT, and acid-fast bacilli. Unfortunately, the next verification did not confirm tuberculosis in PCR (in the same probe acid-fast bacilli were positive), granuloma or mycobacterial staining in lymph nodes The environmental mycobacteria were probable; therefore, treatment was not obligatory. In the next verification still, no cancer cells were observed in the pulmonary lobe nor in lymph nodes.

## 4. Discussion

The immune endocrinopathy shows balance between the pro- (hormonal) and anticancerous (immunity) pathways.

The identified AITD susceptibility genes include immune-modulating genes, such as the Major Histocompatibility Complex (MHC), Cytotoxic T Lymphocyte Antigen-4 (CTLA-4), CD40 molecule, Protein Tyrosine Phosphatase-22, TSH receptor and thyroglobulin (TG). Most of these proteins are responsible for the formation of an immunological synapse, although the role of organ-specific target antigens (structural, unlike thyroid peroxidase) seems to be crucial, since the most important factor in our patients is tissue destruction, not loss of function only.

On the other hand, in NSCLC, immunotherapy with systemic immune checkpoint blockade may be more difficult in the lung niche, because the local microbiome (e.g., CMV) and common infections (like mycobacterial disease) involve lymphocytes and cytokine storms sometimes inducing granulomatous disease as well as specific immuno-escape (for EBV) [12].

### 4.1. Epidemiological, Statistical Versus Clinical Model

Endocrine cancer and immunity predisposition syndromes are inherited entities determined especially by germinal pathogenic variants. Most frequent thyroid cancer predisposition syndromes coexist with endocrinopathy (MEN II, familial adenomatous polyposis) but usually with an immunogenetic background (primary immunodeficiency, Li Fraumeni syndrome, ataxia–telangiectasia) [13]. It is not a full list, because immunodeficiency and immune dysregulation are observed in the destruction of genome integrity, the DNA repair process, miRNA formation and maturation, various signaling pathways as well as predisposition to infectious diseases [2,13]. For example, adenosine deaminase (ADA) may be crucial in specific immunity (mutation causes severe combined immunodeficiency) but in different conditions, such as in patients with tuberculosis, when the ADA level in pleural effusion is high. Our patients showed pleural effusion, which is noteworthy, without cancer cells. Furthermore, thyroid hormone levels change in response to lymphocyte proliferation in patients with tuberculosis and bacterial infections as described previously [14].

A higher incidence of thyroid carcinoma in patients with Graves’ disease was studied in many cohort studies. Correlation is still very controversial, but the crucial issue is about a cause-and-effect relationship, not a statistical one. For example, two retrospective studies using the same methodology showed completely contradictory results: the first clearly indicated a poor prognosis and worse course of TC in patients with GD (including chronicity and recurrence rate) [15,16], the second one indicated the opposite (much smaller tumor size in patients with GD) [5]. The criteria for diagnosing AITD are non-homogenous, and TRAB themselves may be stimulating (TSA) or blocking, which is omitted in cohort studies. Nevertheless, high thyroid hormones do not determine the immune mechanism, as described here (see Table 1), and anti-TG has no defined action as reviewed by Fröhlich et al. [17] However, the first publication is a simple comparison of GD + TC and euthyroid patients with TC [15], but in GD, the athyreosis may also be observed. TC is collectively named differentiated thyroid cancer without subtype.

#### 4.1.1. Bias in Cohort Studies

Both cited publications (as an example) and previously published papers have a basic defect: patients with autoimmune thyroiditis were limited only to GD, and the retrospective analysis included patients with diagnosed TC. Such selection bias is observed and undermines the reliability of statistical studies in the face of contradictory conclusions [18].

The first criterion, i.e., TC, obstructs observation of whether cancer develops more often in patients with thyroiditis (especially GD) and (if yes) after what time. The literature on AITD and TC association is based on preoperative FNAB or post-thyroidectomy histopathology reports, so at the data collection stage, patients are not representative [19]. Conversely, AITD may be an antitumor immunity (the same antigens). Moreover, the presence of antibodies (anti-TPO, anti-TG, or TSA) is very often found in healthy people. The TSA is transitory IgG1 with known effect, which is contrary to anti-TPO and anti-TG [17].

#### 4.1.2. Bias in High Specialization Oncology

Lymph node involvement was found in 56% of patients with Graves’ disease and in 23% of those with toxic goiter, and distant metastases were found in one patient with Graves’ disease only [4].

In our observation, the neck lymphadenopathy was not observed. Contrary to other cancers, the lymphatic system seems to be negligible because the thyroid gland has intensive vascularization. It is noteworthy that the last study showed novel loci containing genes that were previously implicated in thyroid cancer (e.g., HES1, SPATA13, DIRC3, ID4) and a positive association of TSH with VEGFA expression [20], therefore angiogenesis. If we look at our patient with blood-derived metastasis, it is clear that pulmonary circulation is the first filter. Therefore, TNM staging is not a good model for TC, since many TCs are nodule-free and without lymph node involvement.

In 2011, the International Association for the Study of Lung Cancer, American Thoracic Society and European Respiratory Society introduced papillary subtype of lung adenocarcinoma coexisting with very high TTF-1 expression (90–100%) and 5% BRAF mutation [21]. However, BRAFV600E mutation showed a high specificity for papillary TC, especially the classic variant and extrathyroidal extension [22], whereas it was never found in follicular and medullary thyroid carcinoma or in benign thyroid neoplasms. In the high specialization era (according to this classification), our patients (especially the second one) were misdiagnosed with lung adenocarcinoma, since TTF-1 seemed to be the single best marker [23]. The 5% BRAF-positive papillary lung adenocarcinoma might be metastatic TC. Furthermore, the TTF-1 is a protein that regulates the transcription of genes specific for the thyroid, lung, and diencephalon, which is sometimes observed in other carcinomas, e.g., colorectal cancer (see Section 2.2.2 Laboratory Analysis). Therefore, just like tumor markers, IHC and molecular analysis will not replace diagnostic imaging and a holistic approach in deciding on a primary/metastatic tumor in the lung. The publication shows the added value of serving as a pneumocyte marker that can help confirm a primary lung origin. Intriguingly, this “Multidisciplinary Classification of Lung Adenocarcinoma” describes a helpful procedure addressing the question of metastatic adenocarcinoma from the colon or breast but not thyroid gland [21].

Renal cell cancer and lung cancer are the most common cancers metastazing to the thyroid gland. It might also be possible in the case of patient 1. However, this is a less realistic scenario, because no other metastases were observed, thyroid disease significantly preceded the diagnosis of lung cancer, and the patient was a non-smoker. Metastases to the thyroid gland from cancer elsewhere are not considered by most studies based on preoperative FNA or post-thyroidectomy.

Therefore, in clinical observation, TC and NSCLC (adenocarcinoma) symptoms usually overlap, and consequently, differentiation is very difficult. It requires whole body examination (as presented here by PET) and searching for the strict diagnosis of previous endocrinopathy and cancer predisposition syndromes as well as immunodeficiency with DNA sequencing. Patient no. 2 is a good example. Unfortunately, the latest Polish study of genetic predisposition to TC in 1076 unrelated individuals did not describe BRAFV600E [24].

#### 4.1.3. Thyroid Cancer Diagnosis, Reporting, Estimation in Public Health

Thyroid cancer “overdiagnosis” is part of “overmedicalization”, easy empiric therapy, i.e., therapeutic intervention without a final and confirmed diagnosis (usually preemptive therapy). Therapeutic “success” and diagnosis ex juvantibus prompt overreporting TC in public health with financial consequences. Similar discrepancy and reporting, i.e., false statistics, arise wherever treatment is not preceded by thorough diagnostics, as in the three presented cases. All three patients were included in the cancer registry and tumor board decisions but with misdiagnosis [9].

A very similar process, but on a much larger scale, has been observed in the COVID-19 era [25], particularly when we observe global overuse and empiric therapy with antibiotics [26]. For example, hospitalization is finished; the patient was treated according to guidelines with an initial diagnosis of tuberculosis before the final results of mycobacterial culture (it takes 6–8 weeks) (patient 3, finally with mycobacteriosis) or after thoracic surgery before the histopathological result was obtained. The first patient received treatment before the thyroid process was verified. The second patient diagnosed with NSCLC died before the genetic background was analyzed, and the cause of death was not analyzed comprehensively.

Therefore, it is a paradigm that TC progresses slowly, causes symptoms only when advanced, and rarely causes death [27], since the most aggressive and metastatic form (see Section 4.1.2) is classified as lung cancer.

#### 4.1.4. Translational Medicine

Organs such as the prostate, breast, or thyroid gland are not critical (life is possible after complete resection). Best supportive care is not difficult, contrary to lung, pancreatic cancer, etc. Consequently, in the case of TC, it is easier to achieve five-year survival. Chemo-radiotherapy may be difficult when cancer coexists with severe cachexia and infectious processes. Such disease-oriented treatment is prone to errors (like NSCLC instead of TC or mycobacterial process), prolongs progression-free survival (PFS) with OS shortening, and decreases the quality of life, as shown here following the previous tumor board’s decision analysis [9].

Our patients 1 and 2 have short OS as the result of misdiagnosis and infectious process with concomitant medication. Unfortunately, the crucial factor was overall status (WHO) and severe cachexia.

On the other hand, this report sheds light on the underdiagnosis of TC, which is misdiagnosed as TTF-1+ lung adenocarcinoma. As a consequence, the overall survival in TC is good, since many cancerous processes with fast blood-derived spread (without nodule and goiter) were classified as lung cancer; noteworthy, this was with worse prognosis, since the BRAF mutation very specific for TC was classified as NSCLC, papillary type (see above).

#### 4.1.5. Misdiagnosis and False Survival

**Figure 3:**
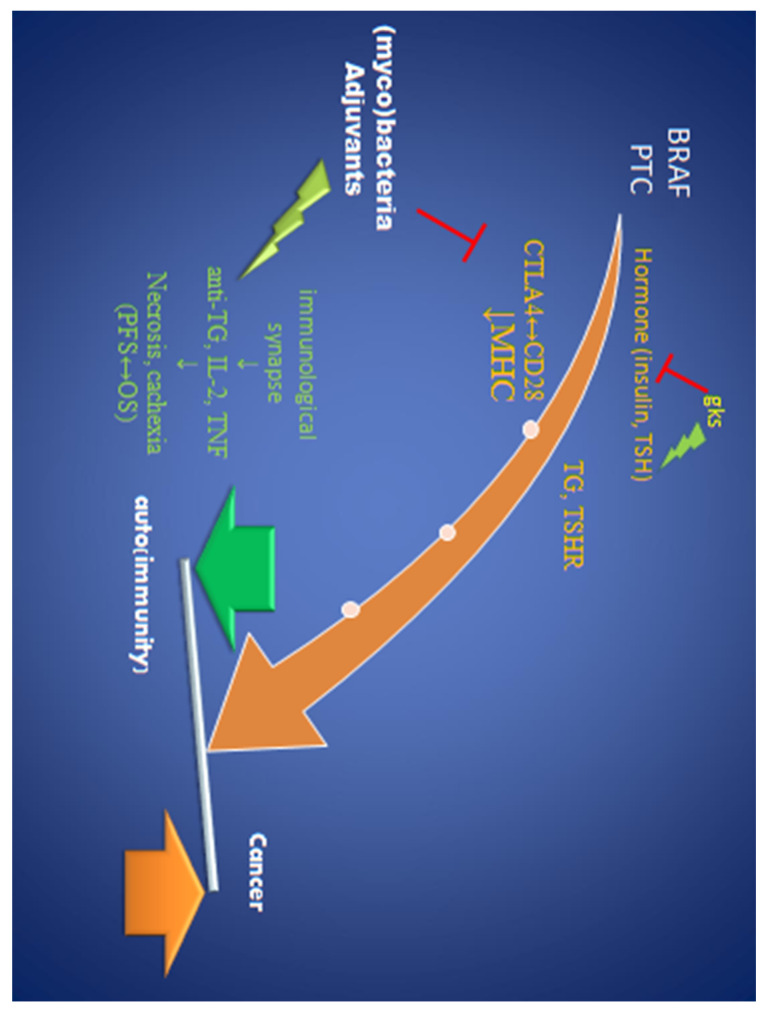
Modification of unique balance between pro- and anticancerous factors (i.e., hormonal and inflammatory signal, respectively) by microbiota (mycobacteria) and steroids. BRAF-BRAFV600E mutation; PTC—papillary thyroid cancer oncogene (RET/PTC), gks—glucocorticoids, MHC—Major Histocompatibility Complex, CTLA4—Cytotoxic T Lymphocyte Antigen-4; TG—thyreoglobulin, TSHR—thyreotropin receptor, TNF—cachectin, PFS—progression-free survival, OS—overall survival. The red symbol indicates the inhibitory effect; the green symbol indicates the stimulating effect.

It is quite a strange phenomenon that cancers with simple observation and biopsy have better prognosis, because they are more likely to be diagnosed in the early stages (statistical increase is also observed). Several bias and overestimations in TC are well-known phenomena in the scientific literature. The increase in TC incidence is a direct consequence of the ease of surgery. Furthermore, histologically proven follicular epithelial dysplasia in the form of scattered microfollicles without colloid and irregularly shaped follicles in autoimmune thyroiditis was observed [27]. FNAB assessment is quite more problematic, and results may be inconclusive in initial oncogenesis, as observed in patient no 3. The pathogenesis of TC in our patients coexisted with the production of proinflammatory cytokines and oxidative stress in autoimmune thyroiditis and tuberculosis. Inflammatory-derived atypia, especially epithelial dysplasia, is usually observed in papillary TC and in a precancerous state as described previously [28]. Interestingly, the same picture in patient 3 corresponded with SUV = 5 in PET despite small volume, low FT4 production, and high TSH, which are the first precancerous hormonal factors (Table 1, Figure 3). 

Higher SUV was observed in mycobacterial inflammation with oxidative stress and the production of proinflammatory cytokines, especially TNF. Currently, another hypothesis considered for the pathogenesis of PTC (papillary TC) in HT is solid cell nest (SCN) [19]. It can be misinterpreted as papillary thyroid microcarcinoma or squamous metaplasia. Contrary to our report and description, SCN is negative for TTF-1 but has strong reactivity for p63.

### 4.2. Clinical Model and Evidence-Based Medicine

Our work is therefore methodologically different (case-control study).

Firstly, it shows the development of the disease over time (Figure 2), i.e., the evolution of crucial parameters (Table 1). Therefore, our observation and very high level of anti-TG indicate for the first time an antigenic target other than the previously indicated one, which is also present in all forms of AITD. It is noteworthy that thyroglobulin is a known TC marker, an extracellular target, observed in various types (Table 1) as described by Fröhlich and Wahl [17]. Therefore, the antigenic stimulation in TC and during inflammatory destruction is higher contrary to an intracellular enzyme (thyroid peroxidase, TPO) or TSH receptor with different expression (low in GD, normalized upon treatment) [20].

Secondly, the aim was to recruit patients in whom the autoimmune process (AITD) preceded the development of cancer (even for years) (Figure 2). Furthermore, we did not limit the influence of AITD to TC only but to broadly understood oncogenesis, since thyroid hormones affect all cells of the body.

Finally, up to now, only childhood exposure to ionizing radiation has been fully recognized as the risk factor, and conflicting results of potential or possible risk factors of TC indicate methodological bias.

Therefore, lessons from individual clinical cases with time-lapse analysis and a comprehensive, holistic approach can be all the more instructive, because the sequence of events is analyzed in a time perspective, i.e., the pathway [29] in vivo. It may be a good example for rare constellation and clinical presentation (many diseases).

### 4.3. Pathway and Time-Lapse Observation

#### 4.3.1. Cachexia: Hormonal Signal and Metabolic Homeostasis

The assessment of nutrition (i.e., effectiveness of nutrient intake in maintaining body composition and meeting metabolic demands) is crucial in the comprehensive medicine and therapy in all patients as presented here in our patients. Although most of the medical textbooks describe nutritional support and energy requirements in patients with very low BMI, the cachexia is a more complex problem than malabsorption. Cachexia is a metabolic syndrome characterized by profound involuntary weight loss. Most of the oncological literature describes cachexia in the light of digestive system cancers (malabsorption in colorectal, deficiency of digestive enzymes in pancreatic cancer). In our patients, cachexia was a result of advanced catabolism related to onco-hormonal and inflammatory disorders. Both of these processes overlap at the systemic (the general symptoms of tuberculosis and thyrotoxicosis are similar) as well as local level, i.e., in the thyroid gland itself as atrophic inflammation (TNF alpha, IL-1, IL-6) with apoptosis and destruction. Triiodothyronine (T3) per se stimulates the apoptosis and protein catabolism as well as the basal metabolic rate, which is crucial for cachexia in our patient. Proinflammatory cytokines production is protease-dependent but also accelerates the proteasome, glycogen synthesis in the liver, and insulin resistance, which were observed in patient 3. The deep proliferation of both cancer cells and competing lymphocytes (in the thyroid gland) and even bacteria (in the lungs) requires enormous amounts of nutrients, especially amino acids. Additionally, protein concentration affects the level of total calcium, but not the ionized one, as seen in patient 2. Finally, in our patients, abnormal thyroid function—i.e., hypo- or hyperthyroidism manifested as myopathy—was observed here in various presentations.

Another underestimated phenomenon is the impact on water management and the action of calcium ions (Table 1). The most common complication after surgery was transient hypocalcemia (36%), which was observed in our patients [30]. Furthermore, the level of calcium as well as of other thyroid hormones (calcitonin) may be useful for the differentiation of TC type. High levels of calcitonin and procalcitonin are typical for MTC (medullary TC [31]). Furthermore, as presented in patient 2, calcinosis may be crucial, since the incidence of TC is higher when local calcinosis is observed [32]. Parathyroid hormone-(PTH)-related protein expressed in various tissues exerts actions by binding to the same type 1 PTH receptor, which regulates calcium homeostasis and white adipose tissue browning with significant lipolysis, fatty acid β-oxidation, and thermogenesis [33]. The PTH is primarily secreted in response to low ionized calcium, which was observed in patients 1 and 3. The hormones are primarily secreted in response to a decrease in ionized calcium, and they physiologically stimulate osteoblast differentiation and bone formation. However, chronic elevation stimulates osteoclast precursor cells [33]. Therefore, there is no simple relationship between calcium metabolism disorders (e.g., calcinosis) and bone destruction, which explains the different picture in our patients (Table 1). A separate element is the use of steroids with osteoporosis, myopathy, and insulin resistance, as observed in patient 3.

BMI at the time of diagnosis was directly related to the risk of thyroid cancer in females [34] but not to OS (presented here).

The role of estrogen in the development of thyroid cancer is still a matter of debate with inconsistent conclusions. The main idea is the observation that TC mainly affects women. If we look at the epidemiological data and the trend in thyroid cancer incidence, we see a similar increase in women and men aged > 65 years, even though earlier AITD affected a vast majority of women [34]. However, this is not a simple relationship. The same is appropriate for autoimmunity, IgM levels, or other X-linked traits, such as the CD154-40 axis, as mentioned among the susceptibility genes, which are key in the differentiation of B lymphocytes [35]. Fetal microchimerism refers to the presence within the maternal organism of a small population of cells originating from the fetus and is proposed as a cause of women’s predisposition [36], but in our observation, there is no difference between multiparous and childless women. Finally, IPEX is X-linked immune dysregulation with autoimmune thyroid disease and opportunistic infections, but, interestingly as in our report, it has an extremely different presentation of hypothyroidism or thyrotoxicosis [37]. Therefore, the final presentation of AITD may be individual, and it is not a simple lack of immune tolerance, as presented here. 

Insulin regulates thyroid gene expression and stimulates thyrocyte proliferation, differentiation, and transformation. Insulin resistance was usually observed in TC females [38]. Hyperinsulinemia, therefore, may be a risk factor for thyroid cancer [35,38]. The key issue is the gks overuse, which, on the one hand, blocks immunization, but it also promotes osteoporosis, insulin resistance, and corticoliberin, which is known as TSH inhibitor [29]. It strictly corresponds with our third case, with high fasting glucose, TSH, and hypocalcemia. Intriguingly, patients under the influence of gks (partly by insulin resistance) develop secondary immunodeficiency with opportunistic infection with non-tuberculous mycobacteria (MOT) (Figure 3). This is an additional target for further research.

The thyroid hormone affects the functioning of nearly all of body organs, and pleural effusion is one of the more severe complications of AITD of note, with very high anti-TPO and anti-TG antibodies, but without TSA [39]. Some authors conducted a literature search and found that kidney dysfunction and hypothyroidism could impact one another. Common coexisting conditions are respiratory failure, heart disease, facial paralysis and pituitary hyperplasia, as well as myopathy with a high non-cardiac creatine kinase level (about 1014 U/L in the presented case). It corresponds with our observations in the first patient (with de Quervain thyroiditis), but the hypocalcemic tetany and cachexia were not described previously. The pleural effusion may correspond with the FT3 and FT4 levels in tuberculosis (FT3 and FT4 levels in patients with effusion were significantly higher). Furthermore, the conversion of T4 to T3 occurs also in the lung, and the activity of type I iodothyronine 5’ deiodinase is statistically significantly lower in lung cancer than in peripheral lung tissue [40]. The paraneoplastic and metabolic consequence of lung adenocarcinoma (especially with high hormonal signal, e.g., TTF-1 expression) may be more systemic and fatal than tumor-related symptoms. Therefore, TNM staging is not adequate for OS and the final prognosis of TC and several lung cancer (e.g., neuroendocrine). Furthermore, cancer and tuberculosis cachexia is a more multifactorial syndrome characterized by progressive weight loss and a disease process that nutritional support cannot reverse. Although advances have been made in animal models of cancer cachexia, translating these advances to the clinic has been challenging, because most of the mouse models do not replicate various cancers [41]. Finally, there is not an animal model of cancer cachexia with severe opportunistic infection such as tuberculosis. In our patients, the crucial pathway seems to be the immunoendocrine axis, promoting proteolysis and catabolism for the inflammatory response and thermogenesis, since the tumor mass (and its energy requirement) was not large.

#### 4.3.2. Various Autoimmunity

It has been suggested that Hashimoto thyroiditis, primary myxedema or AITD, and Graves’ disease are basically similar autoimmune processes with personal diversity and that various clinical presentations depend on individual immunity in specific environmental settings [42]. Basic genetic predispositions are highly polymorphic, and individual genes from the immunoglobulin superfamily, such as major and minor histocompatibility complex (MHC), female sex, and genetic variants of proteins are the antigenic targets of AITD (Figure 3). Unfortunately, statistical studies of TC risk ignore immunogenetic aspects. Although HLA-DR8 and DQB1*0302 are associated with atrophic thyroiditis, it is little understood why only in some people with a specific haplotype the antibodies block the TSH receptor, while in others (GD), they stimulate it. An environmental factor, such as tuberculosis infection, or perhaps medications, seems to be important.

An indirect evidence is the history of patient 3, i.e., with a predominance of the inflammatory process over tumorigenesis and hormonal stimuli. She was not diagnosed with tuberculosis but with non-tuberculous mycobacteria (MOT). It is a self-limiting infection in immunocompetent persons. Importantly, this patient had a very high TSH (Table 1), although high TSH is considered a positive hormonal signal for carcinogenesis. This confirms the unique nature of the immune-endocrine balance (Figure 3).

Furthermore, granulomatous destruction, such as de Quervain thyroiditis, is an extremely rare diagnosis. This disease requires not only antibody testing but also time-lapse observation (Table 1), and the manifestations may be various. The clinical paradigm describes sustained inflammatory response, and in AITD, it may act as a carcinogen. Of note, rare diseases such as de Quervain thyroiditis are not described in the TC context. 

Intriguingly, the in vitro study showed the specific reaction of TSA with TC cells [43].

Interestingly, the observed nodules were not the initial state of oncogenesis, since among cancer-proven patients, about 25% (5/19 pts) were nodule-free and had smaller tumor size and earlier stage TC in comparison to patients without GD [30].

#### 4.3.3. Genetic Background and Immunity

Cassella et al. [30] indicated the advantage of surgery (total thyroidectomy) contrary to radioactive iodine therapy in thyrotoxicosis (e.g., GD), but our observation (case 2) indicates the contrary overbalance in patients with a probability of metastatic TC. On the other hand, the “radical” oncosurgery in NSCLC or TC gives an opportunity for strict genetic diagnosis with an individualized, patient-centered therapeutic regimen. This was missing in the described patients with short OS.

Our case report showed a significant role of immunoendocrinopathy in the presentation and development of extra-thyroid TC. The BRAFV600E mutation shows a high specificity for PTC, especially the classic variant, whereas it was never found in follicular and medullary thyroid carcinoma or in benign thyroid neoplasms [22].

The latest finding showed the role of BRAFV600E mutation, i.e., T > A (V600E), and mutant allele frequency were significantly correlated with tumor size and extrathyroidal invasion without any difference in the pathological lymph node metastasis [44]. It was linked to an increased risk of thyroid capsule penetration, recurrence, and concurrent mutations [45]. Our retrospective analysis confirms the observation. Of note, most of the literature data described lymphadenopathy as a sign of malignancy. Our patients without goiter, significant primary tumors (small nodules only), or neck lymphadenopathy were initially diagnosed by the excision of metastatic focus and misdiagnosed as lung cancer in the clinical picture of these observations (of note, in patient 3, mediastinal lymph nodes were negative for cancer cells and mycobacteria). Despite early radioiodine therapy, the recurrence was observed in our second patient. Our observation showed a timeline with earlier overlooked and ignored symptoms of the immune response against thyroid antigens (especially anti-TG). AITD with tissue destruction preceded the manifestation of cancer for a long time (2–3 years). Concomitant cachexia related to various mechanisms (tuberculosis, intense immune response, thyrotoxicosis and cancer) may be a valuable indicator, because it is a final symptom of these overlapping processes. This is an important indicator for patients with autoimmune thyroiditis, but a common interpretation of GD and TC incidence indicates the tumor-promoting inflammatory mechanism as a paradigm.

However, the negative correlation of GD with tumor size indicates defense forces immunity over tumorigenesis in the initial period of oncogenesis in patients with a deep autoimmune process (Figure 3). It may be a local process only. The unique niche in the lungs (tumor, numerous bacteria and immunomodulatory microbiome) [1,2] creates good conditions for the development of TC elsewhere.

## 5. Conclusions

Numerous statistical studies on the etiology of AITD and TC ignore infectious agents. The only exception is granulomatous (de Quervain) thyroiditis. The temporal association of AITD and TC with mycobacterial infections is quite rare in our observation (three cases out of less than a thousand). In comparison with the prevalence of malignancy in nodular thyroiditis (4–5%), it may indicate a protective role of the inflammatory and autoimmune process, and symptoms may be secondary to substances secreted by the tumor (TG), or they may be a result of antibodies directed against tumors that cross-react with other tissue [46]. In the presented report, critical cachexia, autoimmunity, and osteoarthropathy preceded the discovery of the cancer by weeks to years [46].

Despite numerous studies and statistical correlations, the described potential risk factors of TC coincide with the proposed factors responsible for the increase in all cancer incidence. However, medical intervention must be very careful due to bias (as presented here). The TC incidence growth may be a simple derivative of development and the number of performed procedures because, contrary to other autoimmune diseases, only AITD is treated surgically or with a radioisotope. This, however, does not solve the problem of hidden oncogenesis, since TC rarely metastasizes to lymph nodes, and the lung is the first filter that gives an apparent image of NSCLC as in the presented report. Currently, TTF-1 expression is standard for NSCLC subtype differentiation and NGS for genetic background, but this is not sufficient for pulmonary metastases (especially thyroid and colorectal carcinomas, which are sometimes positive for TTF-1 and BRAF mutation). And the standard should be a strict and holistic diagnosis of all lung cancers in non-smokers, as it is with cancer of unknown origin [47]. Unfortunately, the crucial gap is narrow observation of the chest: a holistic approach and knowledge from various specialties are indispensable.

## Figures and Tables

**Table 1:** Clinical data and outcome of 3 cases with autoimmune thyroiditis, tuberculosis, and thyroid malignancy, initially misdiagnosed as lung cancer. The overlapping presentation is observed: firstly in decreased body weight (cachexia), gender, and age; secondly in lack of neck lymphadenopathy, finally abnormal bone and malabsorption calcium homeostasis and myopathy. Contrary to previous studies, quantitative parameters and their changes over time are shown (evolution is indicated by arrows).

	P1	P2	P3	NORM	
Gender	F	F	F		
Age	67	71	69		
Thyroid disease	De Quervain’s thyroiditis	Graves’ disease	Atrophic thyroiditis		
Initial manifestation	Tachycardia, (FT3/4 fluctuation)	Thyreotoxicosis (exophthalmos)	Hypertension, oedema, insulin resistance		
BMI	20→15	13.4	26.64→22.98		
Neck Lymphadenopathy	no	no	no		
infection	TBC	TBC	MOT		
Overall survival [year]	2.5	3.5	live		
Albumin	3.1	2.8	4.3	3.2–5.0	[g/dL]
Protein	5.0	4.7	6	6.3–8.0	[g/dL]
Uric acid	3.2	5.0	10.0	3.0–7.0	[mg/dL]
Fasting glucose	65	74	153	74–106	[mg/dL]
Procalcitonin	0.08	0.06	0.09		[ng/mL]
Total calcemia	2.32	2.12	1.18	2.2–2.55	[mmol/L]
Ionized calcium	0.82	1.24	0.98	1.15–1.29	[mmol/L]
TSH (during TC diagnosis)	4,33	<0.005	12.13	0.27–4.2	[μIU/mL]
FT4	2.2→1.0	0.96	0.92	0.93–1.7	[ng/dL]
FT3	5.13→2.0	2.43	0.74	2–4.4	[pg/mL]
Anti-TPO	1:320	1:320	47.8 * IU/mL	<1:100	<34 IU/mL
Anti-TSHR	(-)	>1:1280	<0.80 * IU/mL	<1:100	<1.50 IU/mL
Anti-TG	1:1280	>1:1280	984.0 * IU/mL	<1:100	<115 IU/mL
Abnormalities in water/electrolyte balance	pleural effusion hypotonia	hypocalcemia, hypotonia	Oedema		
BONE	Systemic osteopenia	Calcinosis	osteoporosis with pathological fractures		
Dominating process	Various (autoimmunity→cancer)	Various (cancer≈mycobacterial)	mycobacterial		

* Results were presented in quantitative or semi-quantitative (titer) manner for electrochemiluminescence (ECLIA) and immunofluorescence (IF) technique, respectively. Abbreviation: TC—thyroid carcinoma, TPO—thyroid peroxidase, TSHR—receptor for thyro-stimulating hormone, TG—thyroglobulin, TBC—tuberculosis, MOT—non-tuberculous mycobacteria.

## Data Availability

All data supporting the conclusions and describing the course of treatment were included anonymously in the manuscript.
